# Microbiomes of Field-Grown Maize and Soybean in Southeastern and Central Brazil Inferred by High-Throughput 16S and Internal Transcribed Spacer Amplicon Sequencing

**DOI:** 10.1128/MRA.00528-21

**Published:** 2021-08-05

**Authors:** Maike Rossmann, Andrew Maltez Thomas, Suzana Sato Guima, Layla Farage Martins, Patrik Inderbitzin, Victoria Knight-Connoni, Aline Maria da Silva, João C. Setubal

**Affiliations:** a Departamento de Bioquímica, Instituto de Química, Universidade de São Paulo, São Paulo, Brazil; b Indigo Brazil, São Paulo, Brazil; c Department CIBIO, University of Trento, Trento, Italy; d Indigo Ag, Boston, Massachusetts, USA; Indiana University, Bloomington

## Abstract

We report the microbial 16S rRNA gene and internal transcribed spacer (ITS) sequencing data of maize and soybean plants and field soil from eight locations in Brazil. Enterobacter and Pseudomonas were among the most abundant genera. The data suggest the presence of several species that have not been documented for Brazil.

## ANNOUNCEMENT

**FIG 1:**
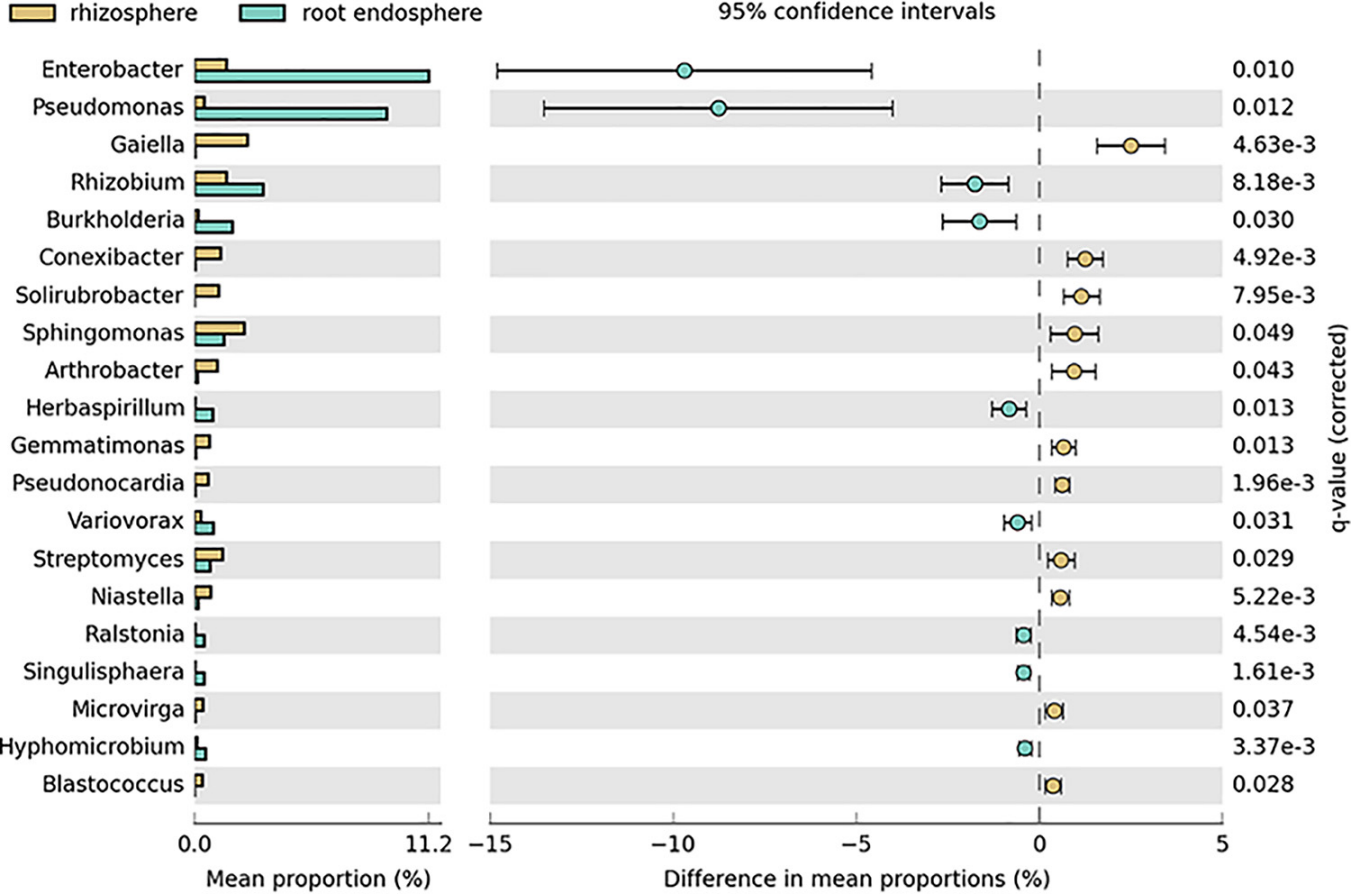
Relative abundance of the most abundant bacterial genera (left) and their differences in mean proportions (right) in the soybean rhizosphere (brown) compared to the soybean root endosphere (blue), based on the 14 rhizosphere and 40 endosphere samples from all sampling sites. The analyses were performed using STAMP v2.1.0 (10). Benjamini-Hochberg-corrected q values, adjusted for multiple comparisons, are provided on the right.

To characterize the fundamental aspects of the maize and soybean microbiomes in the production systems in Brazil, plant and soil samples were collected between March and June 2018 from maize and soybean fields at eight locations in four different states (Table 1). The samples were stored on dry ice immediately after collection. The plant material was surface sterilized before DNA extraction (1). Total DNA was extracted from the plants using the Mag-Bind plant DNA Plus 96 kit (Omega Bio-tek, USA) and from the bulk soil and rhizosphere soil using the DNeasy PowerSoil HTP 96 kit (Qiagen, USA). Rhizosphere soil was removed from roots and placed in a plastic bag through vigorous shaking. The PCR primers were 515F and 806R (2) for the bacterial 16S V4 region and fITS7 (3) and ITS4 (4) for the fungal internal transcribed spacer 2 (ITS2) region. Peptide nucleic acid clamps for the mitochondrial and chloroplast rRNA (5) and fungal mitochondrial rRNA (GCAGACGTGCCCTCG) were used. Two PCR assays were independently performed for each sample and pooled for unique dual-indexing PCR. The amplicon libraries were normalized, pooled (115 to 161 libraries/run), and sequenced on a MiSeq instrument (Illumina, San Diego, CA, USA) using the v3 sequencing kit (500-cycle paired-end 2 × 250-bp sequencing mode) at the Center for Advanced Technologies in Genomics, University of São Paulo, Brazil. The raw reads, 55,440,174 for the 16S sequencing and 55,960,166 for the ITS sequencing, were processed using default parameters unless stated otherwise. The reads were primer trimmed using Cutadapt v1.15 (6) with a maximum 10% mismatch to the primer sequences and processed using DADA2 v1.12 (7). The 16S reads were filtered by allowing a maximum of 2 and 5 expected errors for forward and reverse reads, respectively, and the reads were truncated at the first instance of a base with a quality score less than or equal to 2. In addition, the reads were truncated to a length of 200 bp, and phiX contaminants were removed. Amplicon sequence variants (ASVs) were generated using the trim overhang option, and the chimeras were removed. For ITS, only the forward reads were quality filtered; they were left untruncated for downstream analysis. This resulted in the following numbers of 16S and ITS ASVs, respectively: 4,267/10,653 (leaves), 12,957/12,280 (root endosphere), 68,097/15,536 (rhizosphere), and 53,040/348,923 (bulk soil). The RDP Classifier v2.12 (8) was used to assign taxonomic ranks to bacterial ASVs, and a naive Bayesian classifier (9) trained on the UNITE QIIME database v7.2 for eukaryotes (https://doi.org/10.15156/BIO/587481) was used for fungi. ASVs not classified to at least the phylum level or with ≥95% identity to mitochondrial or plastid sequences were removed. Differences in the relative abundance of taxa between categories were investigated using the Statistical Analysis of Metagenome Profile (STAMP) software v2.1.0 (10). Enterobacter, Pseudomonas, and *Burkholderia* were among the most abundant genera and were significantly more abundant in the root endosphere than in the rhizosphere, in both maize and soybean (Fig. 1). The data suggest the presence of several species in the samples that have not been documented for Brazil at a 99% minimum nucleotide 16S or ITS identity threshold, including Coniochaeta nivea, Curtobacterium gossypii, Mucor brunneogriseus, Pseudomonas glycinis, Sphingomonas aerolata, and Trichoderma paratroviride.

**TABLE 1:** Number of samples collected and sequenced per substrate-crop combination at each location

Location (state: municipality)^*a*^	No. of samples collected from:			
	Leaf endosphere (maize/soy)^*b*^	Rhizosphere soil (maize/soy)^*b*^^,^^*c*^	Root endosphere (maize/soy)^*b*^	Bulk soil (maize/soy)^*d*^
Goiás: Montividiu	5/5	5/4	5/5	5/20
Goiás: Rio Verde	5/5	5/0	5/5	5/20
Mato Grosso do Sul: Uberlândia	5/5	5/0	5/5	5/20
Minas Gerais: Dourados	5/5	5/5	5/5	5/20
Minas Gerais: Maracaju	5/5	5/5	5/5	5/20
Paraná: Imbituva	5/5	5/0	5/5	5/20
Paraná: Lapa	5/5	5/0	5/5	5/20
Paraná: Ponta Grossa	5/5	5/0	5/5	5/20

a A single field was sampled at each location for each crop, except for corn, where bulk soil was sampled from four fields at each location.

b Plant samples from the same substrate were taken from different plants at least 1 m apart and consisted of 2 g of plant material.

c Bulk soil was removed by vigorous shaking in the field.

d Bulk soil samples were taken between rows at a depth of 20 cm at least 1 m apart and consisted of 50 g of soil.

### Data availability.

The raw sequence data have been deposited at the NCBI under BioProject accession number PRJNA524365.
